# Reassessing the efficacy of bevacizumab in newly diagnosed glioblastoma: A systematic review and external pseudodata-based analysis

**DOI:** 10.1093/noajnl/vdad174

**Published:** 2024-01-22

**Authors:** Giacomo Sferruzza, Massimo Malcangi, Luca Bosco, Gaetano Finocchiaro

**Affiliations:** Vita-Salute San Raffaele University, Milan, Italy; Neurology Unit, IRCCS San Raffaele Scientific Institute, Milan, Italy; Vita-Salute San Raffaele University, Milan, Italy; Neurology Unit, IRCCS San Raffaele Scientific Institute, Milan, Italy; Vita-Salute San Raffaele University, Milan, Italy; Neurology Unit, IRCCS San Raffaele Scientific Institute, Milan, Italy; Neurophysiology Unit, IRCCS San Raffaele Scientific Institute, Milan, Italy; Neurology Unit, IRCCS San Raffaele Scientific Institute, Milan, Italy

**Keywords:** bevacizumab, external controls, Kaplan–Meier digitalization, newly diagnosed glioblastoma

## Abstract

**Background:**

First-line use of bevacizumab for glioblastoma (GBM) was evaluated in 2 phase 3 randomized controlled trials (RCT), demonstrating an impact on progression-free survival but not overall survival (OS). However, the crossover events of these trials raised concerns regarding the reliability of this latter analysis. In this study, we conducted an external control-based reassessment of the bevacizumab efficacy in newly diagnosed GBM (ndGBM) against the standard Stupp protocol.

**Methods:**

A systematic review of the literature was conducted to identify the phase 3 RCTs in ndGBM incorporating the Stupp protocol as an arm. For the selected studies, we extracted individual patient survival pseudodata of the Stupp protocol arm by digitizing the Kaplan–Meier plots. A comprehensive pipeline was established to select suitable control studies as external benchmarks.

**Results:**

Among the 13 identified studies identified in our systematic review, 4 studies resulted as comparable with the AVAglio trial and 2 with the RTOG 0825. Pooled individual patient pseudodata analysis showed no differences in terms of OS when bevacizumab was added to the Stupp protocol.

**Conclusions:**

The external-controlled-based reassessment of the bevacizumab treatment in ndGBM confirmed its lack of efficacy in extending OS. Our study includes a summary table of individual patient survival pseudodata from all phase 3 RCTs in ndGBM employing the Stupp protocol and provides a pipeline that offers comprehensive guidance for conducting external control-based assessments in ndGBM.

**Key Points**

- The external control-based analysis confirms the lack of beneficial effect of bevacizumab in ndGBM.
- The individual patient survival pseudodata from all the published Stupp protocol arms are provided for future application.

**Importance of the Study**

Glioblastoma (GBM) is the most common malignant primary brain tumor with a poor prognosis, despite the standard radio/chemotherapy regimen known as the Stupp protocol. In 2 phase 3 clinical trials, bevacizumab failed to prolong overall survival (OS) when added to the standard protocol. However, concerns have arisen regarding this result due to the crossover events of the trials. To address this, we collected all the trials conducted in newly diagnosed GBM (ndGBM) and reconstructed the individual patient pseudodata from the Stupp protocol arms. This allowed us to conduct an external-controlled reassessment of the bevacizumab treatment, confirming its failure in prolonging OS in this setting. To the best of our knowledge, this is the first study to collect and publish all the individual patient survival pseudodata from the standard protocol assessed in a clinical trial setting in ndGBM. Along with a baseline matching pipeline, this data is now available for future external-controlled trials.

Glioblastoma (GBM) is a rare primary brain cancer with an incidence of 3.2 new cases per 100 000 persons per year.^1^ The expected 5-year survival rate is 4.9%, making GBM one of the most aggressive cancers. The standard treatment for newly diagnosed GBM (ndGBM), known as the Stupp protocol, is still based on a 2005 phase 3 study. It involves surgical debulking and radiotherapy with concomitant chemotherapy using the alkylating agent temozolomide (TMZ), followed by adjuvant TMZ.^2^ The most crucial predictive marker for TMZ response is the methylation status of the promoter region of the MGMT gene. In most patients, methylation is absent, resulting in little to no benefit from TMZ chemotherapy.^3^ During the 20 years following the publication of the Stupp protocol, several therapeutic strategies were investigated to improve outcomes for these patients, with disappointing results. Except for tumor-treating fields, none of these approaches demonstrated clear superiority over the standard Stupp Protocol.^4^ Among these strategies, bevacizumab created great hope among neurooncologists due to the dramatic and unequivocal radiological responses observed in pivotal uncontrolled phase 2 trials.^5,6^ By binding to circulating vascular endothelial growth factor A (VEGF-A) and altering its interaction with its receptor on endothelial cells, bevacizumab is able to downregulate angiogenesis.^7^ This mechanism provided a strong rationale for considering bevacizumab as a therapeutic option in highly vascularized tumors like GBM.^8^ Despite this, 2 large phase 3 randomized controlled trials (RCTs) evaluating bevacizumab in ndGBM failed to demonstrate improvement in overall survival (OS) compared to the Stupp protocol.^9,10^ However, the crossover events in these 2 trials have given rise to concerns regarding the analysis of OS. In fact, in the RTOG 0825 trial,^9^ 48% of participants in the Stupp protocol arm-initiated bevacizumab after experiencing progression. Similarly, in the AVAglio trial,^10^ 31% of controls switched to bevacizumab after progression. As a result, the interpretation of the OS analysis of these trials has been challenging. This methodological issue exemplifies a relatively common challenge in clinical trials within neuro-oncology since the notably dire prognosis of patients frequently necessitates a crossover design for ethical considerations. Furthermore, given that the most promising immunotherapy approaches in neuro-oncology involve invasive and biologically costly procedures, such as leukapheresis^11^ or hematopoietic stem cell collection (clinicaltrials.gov: NCT03866109), which are also required in the control arm for a properly controlled blinded design, the availability of crossover emerged as essential to motivate patient enrollment. All in all, the use of experimental therapy as a crossover can complicate OS analysis. This issue is particularly relevant for therapies such as bevacizumab, for which an alternative endpoint like progression-free survival (PFS) is more challenging to interpret due to its known effect on blood–brain barrier stabilization.^12^

A potential solution to this issue is to use external controls.^13,14^ The utilization of external control populations brings about significant methodological challenges that require careful evaluation to avoid misleading interpretations. The field of GBM, specifically, has directly experienced a big disappointment after the drug development pipeline of Rindopepimut, where 3 Phase 2 externally controlled trials^15–17^ provided positive results, leading to the decision to proceed with a phase 3 trial that failed to confirm the beneficial effect of this approach. A similar trajectory can be outlined for the use of nivolumab in recurrent GBM, where a phase 2 clinical trial demonstrated an effect in prolonging OS compared to historical control.^18^ However, the large phase 3 CheckMate 143 did not show any effect.^19^ In this paper, we address this methodological concern by compiling a dataset of individual patient pseudodata (IPP) from the Stupp protocol arms of all the phase 3 clinical trials conducted in ndGBM obtained by a systematic review of the literature. Furthermore, we establish a systematic approach for selecting the most suitable controls for analysis based on known prognostic factors. We conclude our study by presenting a comprehensive database that encompasses the individual patient survival pseudodata of the Stupp protocol arms serving the purpose of conducting an external control-based overall survival analysis in ndGBM.

## Materials and Methods

### Identifying Phase 3 Clinical Trials for ndGBM

To conduct an external control analysis, we decided to focus on phase 3 clinical trials only. This choice aligns with FDA guidelines for control groups in clinical trials (https://www.fda.gov/media/71349/download), which specify that an external control arm data source necessitates sufficient individual patient-level data to ensure a well-powered comparison.

Therefore, we conducted a systematic review of the literature following the recommended guidelines outlined in the Preferred Reporting Items for Systematic Reviews and Meta-Analyses (PRISMA) statement^20^ (see Supplementary Materials 2). Specifically, a comprehensive search for peer-reviewed articles written in English was conducted across databases including PubMed, Scopus, Embase, and Web of Science, aiming to identify phase 3 clinical trials involving patients diagnosed with ndGBM. The search spanned from January 2003 to July 2023. Further details about the search approach can be found in the supplementary methods.

Reference lists of the included studies were also screened. Studies were considered for inclusion if they met the following criteria: a phase 3 clinical trial with an RCT design, enrollment of adult patients (≥18 years) with ndGBM, and incorporation of the Stupp protocol as either a comparator or a treatment arm. Only trials that reported OS outcomes were included. During the review process, the references of the included studies were meticulously screened. Two reviewers (M.M. and L.B.) independently assessed the abstracts of all identified papers and selected those that met the inclusion criteria. In cases where discrepancies arose, a third reviewer (G.S.) was consulted to solve the issue.

The Revised Cochrane risk-of-bias tool for randomized trials (RoB 2) was used to assess the quality and risk of bias of the included studies.^21^ The assessment was conducted independently by L.B. and M.M., with any disagreement resolved through consensus among G.S., M.M., and L.B.

### Data Extraction

For each of the included studies, the demographic and baseline clinical characteristics of the arm treated with the Stupp protocol were independently extracted by the 2 authors (M.M. and L.B.). Extracted data encompassed the known prognostic factors for survival in adult patients with GBM.^22–24^ Specifically: gender prevalence, mean age and its SD, KPS, and the methylation status of the MGMT promoter, along with the percentage of patients evaluated for this molecular marker, the prevalence of gross total resection and the exclusion of patient because of early disease progression. If one of these data was not reported in the study, the corresponding author was contacted. If a response was not obtained, data was estimated according to the following procedure.

Regarding patient age, in cases where means and SD were not explicitly reported, we estimated these values using the median and interquartile range or range, as described in the method outlined by Wan et al.^25^ Furthermore, the same pipeline was applied to the bevacizumab-treated arm of the RTOG 0825 and AVAglio (Figure 2) trials.

**Figure 1.**
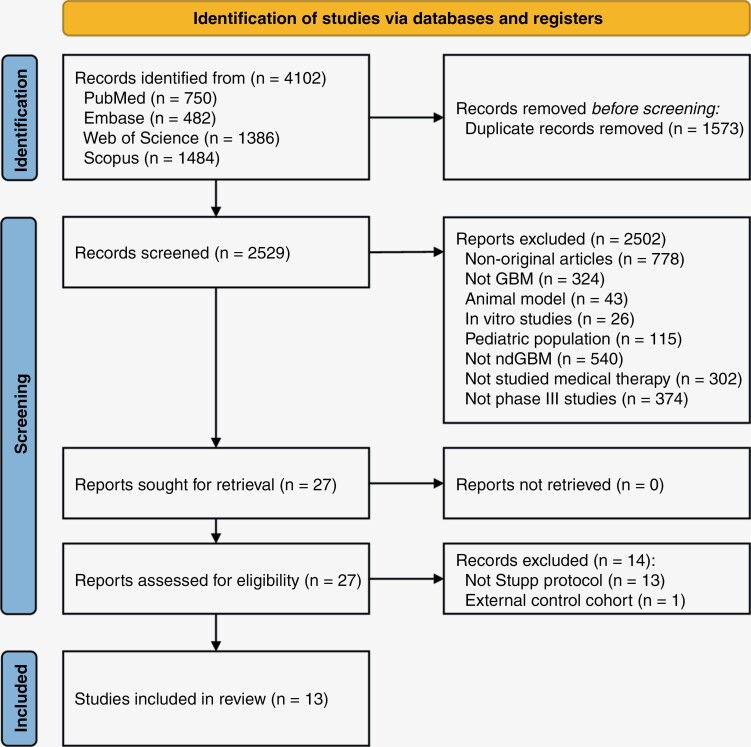
Flowchart of systematic search and study selection, adapted from PRISMA flow diagram.^47^ PRISMA = Preferred Reporting Items for Systematic Reviews and Meta-Analyses, GBM = glioblastoma, ndGBM = newly diagnosed glioblastoma.

**Figure 2.**
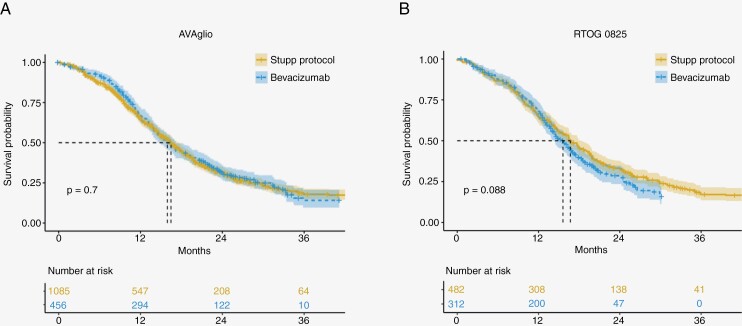
Overall survival of patients treated with bevacizumab compared to matched external controls. The bevacizumab (dashed line) arms of the AVAglio (a) and RTOG 0825 (b) trials were compared to the Stupp protocol arms (solid line) of the trials, which were selected as external controls based on their clinical and demographic characteristics. Median survival times are indicated by the black dotted lines, and the 95% CI is depicted in the shaded area.

To reconstruct individual patient survival pseudodata from Kaplan–Meier (KM) curves for OS, we digitally scanned the published KM curves using the freely accessible web tool WebPlotDigitalizer v. 4.6. Subsequently, we reconstructed individual patient pseudodata using the R package “IPDfromKM” (R Foundation for Statistical Computing, Vienna, Austria, version 4.0.3), which implements the modified iterative KM estimation algorithm proposed by Guyot et al.^26^

KM digitalization was performed by M.M. and G.S., while data reconstruction was carried out by G.S. using version 4.0.3 of the R statistical package (R Foundation for Statistical Computing, Vienna, Austria). The script of the analysis conducted is reported in supplementary materials.

To evaluate the accuracy of the method, the median OS of the reconstructed curves was compared to the data reported in the original studies.

### Control Selection Pipeline and Statistical Analysis

The inability to manage bias remains the primary challenge in externally controlled trials. Nevertheless, within this experimental context (ie assessing the impact of a drug on OS in ndGBM), we are in a favorable position to mitigate such bias according to the FDA guidelines for control groups in clinical trials (see https://www.fda.gov/media/71349/download). Specifically, our study endpoint is inherently objective, patients received uniform treatment and evaluation in a trial setting and most of the covariates known to affect the outcomes were comprehensively characterized and reported as summary statics in the included studies. It should be noted, however, that the analysis used in this study is based on individual patient pseudodata extracted from KM curves. This method does not allow for patient-level control of covariates. Instead of this approach, an evaluation of summary statistics of covariates provided in the included studies was used to match the most appropriate controls.

In this scenario, the 6 demographic and baseline clinical criteria (age, sex prevalence, KPS, MGMT methylation status, gross total resection, and the exclusion of early progression) can be compared using Chi-square or *t*-tests as suitable, to select the most closely matched external control. The sample size of phase 3 clinical trials often makes it possible to detect small differences in patient age, which may bear little relevance given the nonlinear relationship between age and GBM prognosis.^27^ For this reason, we considered significant a difference in patient age of more than 5 years. Furthermore, we treated missing data as discrepancies.

Despite the use of corticosteroids at baseline being demonstrated as an independent prognostic factor in GBM patients,^28^ we failed to include this data in our study since it was frequently unreported in the included studies.

To select the most appropriate control populations, we compared summary statistics of the 6 aforementioned prognostic factors. We accepted a maximum of 2 prognostic factor discrepancies, except for MGMT promoter methylation status because of its strong impact as a prognostic factor.^22,24^ However, future studies that may utilize our database and methods for different purposes, should carefully select the threshold for discrepancy and, consequently, the potential risk of bias. This consideration should be aligned with the specific goals of the study. For instance, we highly recommend employing a higher threshold, ideally to zero prognostic factor discrepancy, when utilizing external controls in phase 2 clinical trials evaluating the feasibility of progression to a phase 3 clinical trial.

A relevant pitfall of this approach is that we do not have information on the number of patients enrolled in the included clinical trials who received bevacizumab after disease progression.

So, to avoid a further “crossover effect” in the external control analysis, we conducted a secondary analysis including only the clinical trials that had concluded patient follow-up before FDA approval of bevacizumab in 2009.^29^

Once the selected external controls were identified, individual patient pseudodata were pooled to form the control arms and compared to the bevacizumab arms of the AVAglio and RTOG 0825 trials using a 2-sided log-rank test. Leave-one-sensitivity analysis was performed to evaluate the consistency of the results and identify potential sources of heterogeneity.

## Results

### Search Results and Characteristics of the Included Studies

As summarized in Figure 1, our search initially identified a total of 4102 records, of which 13 met the inclusion criteria for this systematic search.^2,4,9,10,30–38^ The estimated risk of bias was low for all the included studies except for 2^32,35^ because of deviations from the intended intervention. In total, the Stupp protocol arms of the included studies enrolled 3522 patients. The clinical and demographic characteristics of the included studies are reported in Table 1. The methylation status of the MGMT promoter (reported in Table 1) displayed a range of variability among the included studies. One trial exclusively enrolled unmethylated patients, 3 trials included only methylated patients, 1 trial had an almost balanced proportion, and 8 trials predominantly enrolled unmethylated patients, mirroring the prevalence of this molecular feature in real-life scenarios. The study of Stupp et al. (2005),^2^ represents the first trial in which the relevance of MGMT promoter methylation status was demonstrated in determining the response to TMZ**.** This data was presented in the subsequent benchmark publication for a subgroup of patients in whom MGMT promoter methylation status could be determined, representing 37.1% of the Stupp protocol arm.^3^ In this subgroup MGMT promoter resulted methylated in 43.3% of the patients. One study^36^ did not report any information regarding the methylation status of the MGMT promoter, even in the subsequent published post hoc analysis based on pathological data.^39^ This omission renders it unsuitable for our pipeline.

**Table 1.** Baseline Characteristics and Median Overall Survival of the Stupp Protocol Arms in the Included Studies

Author (Year)	*N*	Sex (Male%)	Age (Mean ± SD)	KPS ≥ 90 (%)	Evaluated for MGMT status (%)	Methylated MGMT (%)	Gross Total Resection (%)	Excluded Early Progressions	Median OS
Stupp et al. (2005)	287	64.5	50.2 (±8.9)^a^	39.4	37.1	43.4	39.4	No	14.6
Gilbert et al. (2013)	411	58.2	55.0 (±10.5)^a^	66.4	91.5	32.4	56.0	Yes	16.6
Chinot et al. (2014)	463	64.4	52.3 (±10.2)^a^	69.7	76.9	33.7	42.3	Yes	16.7
Gilbert et al. (2014)	309	62.8	53.8 (±11.0)^a^	61.9	96.8	28.4	60.3	Yes	16.1
Stupp et al. (2014)	273	52.4	60.7 (±3.0)^a^	55.5	100.0	100.0	50.6	Yes	26.32
Westphal et al. (2015)	71	63.4	53.0 (±8.4)	NA	67.6	33.3	42.3	No	19.6
Kong et al. (2017)	89	57.3	55.8 (±10.5)	NA	0.0	NA	53.9	Yes	16.88
Stupp et al. (2017)	229	68.6	53.3 (±11.0)^a^	66.8	75.1	44.8	53.7	Yes	16
Weller et al. (2017)	374	61.0	58.0 (±8.9)^a^	44.9	93.0	37.4	56.1	No	17.4
Herrlinger at al. (2019)	63	47.6	58.3 (±10.6)^a^	77.8	100.0	100.0	63.5	No	31.4
Lim et al. (2022)	358	55.0	54.8 (±10.8)^a^	70.3	99.4	98.0	55.9	No	32.1
Lassman et al. (2023)	316	59.5	57.8 (±9.2)^a^	62.7	100.0	37.0	57,0.8	No	18.7
Omuro at al. (2023)	280	62.5	54.0 (±10.2)^a^	75.4	100.0	0.0	51.4	No	14.9

^a^Data were estimated using the median and interquartile range or range reported in the studies.^21^ Age is reported in years, the median time from the initial diagnosis to randomization and OS is reported in months. Abbreviations: *N* = number of patients, OS = overall survival, NA = not available.

A broad spectrum of median OS was noted within the encompassed studies, spanning from 14.6 to 32.1 months. Notably, all studies reporting a median survival exceeding 20 months exclusively enrolled MGMT methylated patients. Baseline patient characteristics, along with the median OS of the bevacizumab arms in the RTOG0825 and AVAglio trials are reported in Table 2.

**Table 2.** Baseline Characteristics and Median Overall Survival of the Bevacizumab Arms in the RTOG and AVAglio Trials

Characteristic	Chinot et al. (2014) (*N* = 458)	Gilbert et al. (2014) (*N* = 312)
Sex (male%)	61.6	57.1
Age (mean ± SD)	54.5 (±10.7)^a^	55.3 (±10.6)^a^
KPS ≥ 90 (%)	67.2	60.3
Evaluated for MGMT status (%)	74.7	97.8
Methylated MGMT (%)	25.5	28.8
Gross total resection (%)	41.1	35.2
Excluded early progressions	Yes	Yes
Median OS	16,8	15,7

^a^Data were estimated using the median and interquartile range or range reported in the studies.^21^ Age is reported in years, the median time from the initial diagnosis to randomization and OS is reported in months.

Abbreviations: *N* = number of patients, OS = overall survival, NA = not available.

The OS IPP obtained from the Stupp protocol arm of the included studies are reported in the Supplementary Table 1. Supplementary Table 3 provides the median OS and 95% confidence interval (CI) derived from the reconstructed data and as reported in the original paper. These variations are documented in each section and consistently remain within a 3% difference (as shown in Supplementary Table 1). To further demonstrate the consistency of our analysis, we also reported in Supplementary Table 3 the 2-year survival rate derived from our analysis and as reported in the paper.

### Evaluation of Bevacizumab Efficacy in ndGBM

According to the pipeline described in the method sections, 4 Stupp protocol arms were deemed suitable as external control groups for the AVAglio trial^2,31,37,38^ (see Table 3), and 2 for the RTOG 0825 trial^31,37^ (Table 4). These studies were selected as external controls because they showed discrepancies for a maximum of 2 prognostic factors and did not show significant differences in the proportion of patients with MGMT promoter methylation. We pooled the individual patient pseudodata from the selected studies, creating 2 Stupp arms with 1085 and 482 patients, respectively. Upon reevaluating the treatment arm of the AVAglio trial and RTOG 0825 with their respective external controls, no significant differences emerged in overall survival. Specifically, for the AVAglio trial, a median overall survival (mOS) of 15.9 months (95% CI: 14.8–17.2) was calculated, while it was 16.5 (95% CI: 15.6–17.7) for the control group (*P*-value = .7). The 2-year survival rate was 31.22% (95% CI: 27.1–35.9) for the bevacizumab arm and 31.5% (95% CI: 28.4–34.9) for the control group.

**Table 3.** Comparison of Patient Characteristics Among AVAglio Treatment Arm and Stupp Protocol Arms in the Included Studies

Chinot et al. (2014) (AVAglio)							
	Sex	Age	KPS	Methylated MGMT	Gross Total Resection	Excluded Early Progressions	Risk of Bias
Stupp et al. (2005)	0.54	<0.01	≤0.01	0.09	0.65	X	☺
Gilbert et al. (2013)	0.30	0.49	0.76	0.62	0.01	✓	☺
Stupp et al. (2014)	0.01	≤0.01	≤0.01	≤0.01	0.01	✓	☹
Westphal et al. (2015)	0.77	0.26	NA	0.90	0.85	X	☺
Kong et al. (2017)	0.45	0.29	NA	NA	0.30	✓	☹
Stupp et al. (2017)	0.07	0.17	0.59	0.02	≤0.01	✓	☹
Weller et al. (2017)	0.85	< 0.01	≤0.01	0.39	≤0.01	X	☹
Herrlinger at al. (2019)	0.03	0.01	0.10	≤0.01	0.02	X	☹
Lim et al. (2022)	0.06	0.69	0.37	≤0.01	≤0.01	X	☹
Lassman et al. (2023)	0.34	<0.01	0.17	0.45	≤0.01	X	☺
Omuro at al. (2023)	0.80	0.71	0.02	≤0.01	≤0.01	X	☹

*P*-values refer to *t*-test for continuous variables and the Chi-squared tests for categorical ones. Significant *P*-values (*P* < .05) are indicated by underlining, except for Age, for which only differences ≥5 years were considered clinically relevant. The matching of the exclusion of early progression criteria was also evaluated.

Abbreviations: *N* = number of patients, OS = overall survival, NA = not available, ☺= low risk of bias; ☹= potential risk of bias.

**Table 4.** Comparison of Patient Characteristics Among RTOG Treatment Arm and the Stupp Protocol Arms in the Included Studies

Gilbert et al. (2014) (RTOG 0825)							
	Sex	Age	KPS	Methylated MGMT	Gross Total Resection	Excluded Early Progressions	Risk of Bias
Stupp et al. (2005)	0.09	≤0.01	≤0.01	≤0.01	0.29	X	☹
Gilbert et al. (2013)	0.09	0.70	0.09	0.41	≤0.01	✓	☺
Stupp et al. (2014)	0.26	≤0.01	0.25	≤0.01	≤0.01	✓	☹
Westphal et al. (2015)	0.33	0.09	NA	0.59	0.28	X	☺
Kong et al. (2017)	0.97	0.69	NA	NA	≤0.01	✓	☹
Stupp et al. (2017)	≤0.01	0.03	0.12	≤0.01	≤0.01	✓	☹
Weller et al. (2017)	0.30	< 0.01	≤0.01	0.03	≤0.01	X	☹
Herrlinger at al. (2019)	0.17	0.04	≤0.01	≤0.01	≤0.01	X	☹
Lim et al. (2022)	0.60	0.55	≤0.01	≤0.01	≤0.01	X	☹
Lassman et al. (2023)	0.53	< 0.01	0.54	0.05	≤0.01	X	☹
Omuro at al. (2023)	0.18	0.56	≤0.01	≤0.01	≤0.01	X	☹

*P*-values refer to the *t*-test for continuous variables and the Chi-squared tests for categorical ones. Significant *P*-values (*P* < .05) are indicated by underlining, except for Age, for which only differences ≥ 5 years were considered clinically relevant. The matching of the exclusion of early progression criteria was also evaluated.

Abbreviations: *N* = number of patients, OS = overall survival, NA = not available, ☺= low risk of bias; ☹= potential risk of bias.

For the RTOG 0825 trial, a mOS of 15.6 months (95% CI: 14.4–16.9) was reconstructed, compared to 16.7 (95% CI: 15.5–18.2) of the control group (*P*-value = .09). The 2-year survival rate was 28.6% (95% CI: 23.5–34.8) compared to 33.0% (95% CI: 28.9–37.6) for the control group.

Sensitivity analysis conducted by means of leave-one-out analysis demonstrated substantial stability of the pooled analysis (see Supplementary Table 2), except in the external control analysis of RTOG 0825 after the removal of Gilbert et al. (2013), in which the bevacizumab arm displayed reduced survival compared to the Stupp protocol arm of Westphal et al. (2015). Furthermore, in the secondary analysis, when comparing patients treated with bevacizumab in the AVAglio trial with the single study^2^ that concluded follow-up before FDA approval of bevacizumab in 2009,^29^ no significant difference in overall survival was noted (*P*-value = .12). We did not perform the same analysis for RTOG 0825 due to the higher risk of bias.

## Discussion

This study started to address a specific question: whether the failure of bevacizumab in prolonging OS of GBM patients was due to the crossover events of the AVAglio and RTOG 0825 trials. This hypothesis was introduced the year following the trial publication^40^ and found support in 2 subsequent observations: (1) the randomized phase 2 trial BELOB,^41^ conducted in the Netherlands where bevacizumab was unavailable, minimizing crossover effects, reported improved OS in recurrent GBM when bevacizumab was added to lomustine compared to either single therapy, and (2) a post hoc analysis of the AVAglio trial demonstrated that in the subgroup of patients who did not receive therapy after confirmed progression (thus excluding all crossover possibilities), bevacizumab extended both PFS and OS.^42^ Despite the limitations inherent in such analyses, these 2 pieces of evidence raised legitimate doubt about the conclusions of these 2 trials. This scenario is not uncommon in oncology trials, where the crossover design can be dictated by ethical considerations or the randomized controlled design can be unfeasible due to financial or organizational constraints.^13^ Different approaches can be used to overcome this limitation and evaluate the OS in the context of a crossover design, including rank-preserving structural failure time models^43^ or inverse probability of censoring weighting models.^44^ However, these methods require working with individual patient data that crossed over to the experimental treatment, which we did not have in this specific case. So, starting from this limitation, we adopted a methodology similar to that utilized in a recent trial published by Liau et al.^11^ that can be suitable for exploratory analysis even when a control group is not included in the trial design at all, as is often the case in phase 2 trials.

In the clinical trial, Liau et al. evaluated the efficacy of an autologous tumor lysate-loaded dendritic cell vaccine, and a crossover design was deemed essential for reasons of feasibility and ethics. For this reason, as prespecified in the statistical analysis plan approved by the regulatory institutions, the author selected the most closely matched external control populations to analyze OS.

Similarly, in the present study, we conducted an external control reassessment of the 2 trials evaluating bevacizumab in ndGBM, selecting the external controls among the studies identified through a systematic literature review of all the phase 3 clinical trials in ndGBM that included an arm treated with the Stupp protocol. The results of this analysis did not unveil a significant effect of bevacizumab on OS in ndGBM.

As a result of our systematic review, we incorporated a summary table in this study containing all the IPP from the arms treated with the Stupp protocol across all phase 3 trials ever conducted in ndGBM. Additionally, we included a summary table featuring the collected baseline prognostic factors, aimed at assisting in the selection of the most appropriate external control populations for future applications. This pool of potential external control populations encompasses a comprehensive spectrum of prognostic factor combinations, with special emphasis on the MGMT methylation status. Furthermore, if this database will be employed to conduct exploratory analysis in a new trial lacking internal controls, the authors could implement a matching-adjusted indirect comparison to the treated arm as outlined in,^45^ thus enabling further adjustments for minor disparities in individual patient characteristics.

Our study is not exempt from limitations. The primary intrinsic limitation in using reconstructed survival pseudodata from KM curves is that this approach does not permit covariate control at the individual patient level. Patient-based covariates are only retrievable as summary statistics from the original paper. This discrepancy allows for covariate control only at the population level, rather than at the individual patient level and limits the possibility to perform more specific sensitivity analysis. The second significant limitation is that we lacked information on the number of patients in the control groups who switched to bevacizumab after disease progression. This introduces the possibility of a “crossover effect” that cannot be quantified in our analysis. To address this limitation, we conducted a secondary analysis using data from the AVAglio trial. In this analysis, we employed the initial study that investigated the efficacy of the Stupp protocol as an external control, as it concluded prior to the FDA’s approval of bevacizumab. This additional analysis also produced negative results.

Another limitation concerns the definition of gross total resection, which varied across the included studies, potentially introducing bias. In addition, the use of corticosteroids at baseline, a factor known to represent an independent prognostic factor,^28^ was not included in this study due to the frequent unavailability of this data in the included studies. Moreover, the 2021 update of the World Health Organization (WHO) diagnostic criteria for the CNS^46^ introduced notable changes in glioma classification, identifying IDH-mutant forms of GBM as a distinct entity termed “diffuse astrocytoma IDH-mutant.” While this potential limitation did not impact the current study, given that all the enrolled studies recruited patients prior to the publication of this revised classification, it is a crucial aspect to consider for future applications of these datasets. Furthermore, it must be considered that the known prognostic factors included in the study, as well as those known but not included such as corticosteroids, can only serve to mitigate the potential risk of selection bias, which can be adequately addressed only through the use of a randomized design. Consequently, the strategy outlined here should not be viewed as a substitute for a randomized and blinded design but rather as a secondary option to be pursued only when the latter approach proves unfeasible. Despite these limitations, we believe that in cases where a randomized controlled design is deemed unfeasible, the methodological approach we described, along with the individual patient pseudodata made available in this paper, provides a useful methodology to conduct exploratory analysis, leveraging the wealth of available data retrieved from past clinical trials.

In conclusion, our reassessment of the AVAglio and RTOG 0825 trials, conducted through a comparison with external control populations, did not reveal a beneficial effect on OS resulting from the addition of bevacizumab to front-line radio/chemotherapy GBM. The extensive dataset we have presented, encompassing the individual patient survival pseudodata from the standard Stupp protocol arm in each trial conducted in ndGBM, along with the outlined methodology for selecting appropriate external controls, could serve as a benchmark for conducting externally controlled trials in this patient population.

## Supplementary Material

vdad174_suppl_Supplementary_Data

vdad174_suppl_Supplementary_Tables_S1-S3

## Data Availability

All relevant data used to conduct this study are published in the main text or supplementary files, except for the list of records identified by the systematic search, which will be made available upon reasonable request.
