# Supplementary material for: Reassessing the efficacy of bevacizumab in newly diagnosed glioblastoma: A systematic review and external pseudodata-based analysis
Source: Neurooncol Adv. 2024 Jan 22;6(1):vdad174. doi: 10.1093/noajnl/vdad174 (PMC10883711; doi:10.1093/noajnl/vdad174)
Supplement: vdad174_suppl_Supplementary_Tables_S1-S3 [file vdad174_suppl_supplementary_tables_s1-s3.docx]

**Search strategy**

The search strategy in Pubmed was: ((glioblastoma) OR (glioblastoma[MeSH Terms])) AND ((phase 3) OR (phase III)) AND (clinicaltrial[Filter]) AND (2003:2023[pdat]) AND (english[Filter])

The search strategy in Scopus was: TITLE-ABS-KEY ( glioblastoma ) AND TITLE-ABS-KEY ( clinical AND trial ) AND TITLE-ABS-KEY ( ( phase 3 ) OR ( phase AND iii ) ) AND PUBYEAR > 2002 AND ( LIMIT-TO ( DOCTYPE , "ar" ) ) AND ( LIMIT-TO ( LANGUAGE , "English" ) ) AND ( LIMIT-TO ( EXACTKEYWORD , "Human" ) )

The search strategy in Embase was: ('glioblastoma'/exp OR glioblastoma) AND ((phase AND 3) OR (phase AND iii)) AND [2003-2023]/py AND 'clinical trial'/de AND 'human'/de AND 'article'/it

The search strategy in Web of Science was: ALL=(glioblastoma) AND ALL=(clinical trial) AND ALL=((phase 3) OR (phase III)) and 2023 or 2022 or 2021 or 2020 or 2019 or 2018 or 2017 or 2016 or 2015 or 2014 or 2013 or 2012 or 2011 or 2010 or 2009 or 2008 or 2007 or 2006 or 2005 or 2004 or 2003 (Publication Years) and Article (Document Types)

**Quality assessment – Table S1**

**
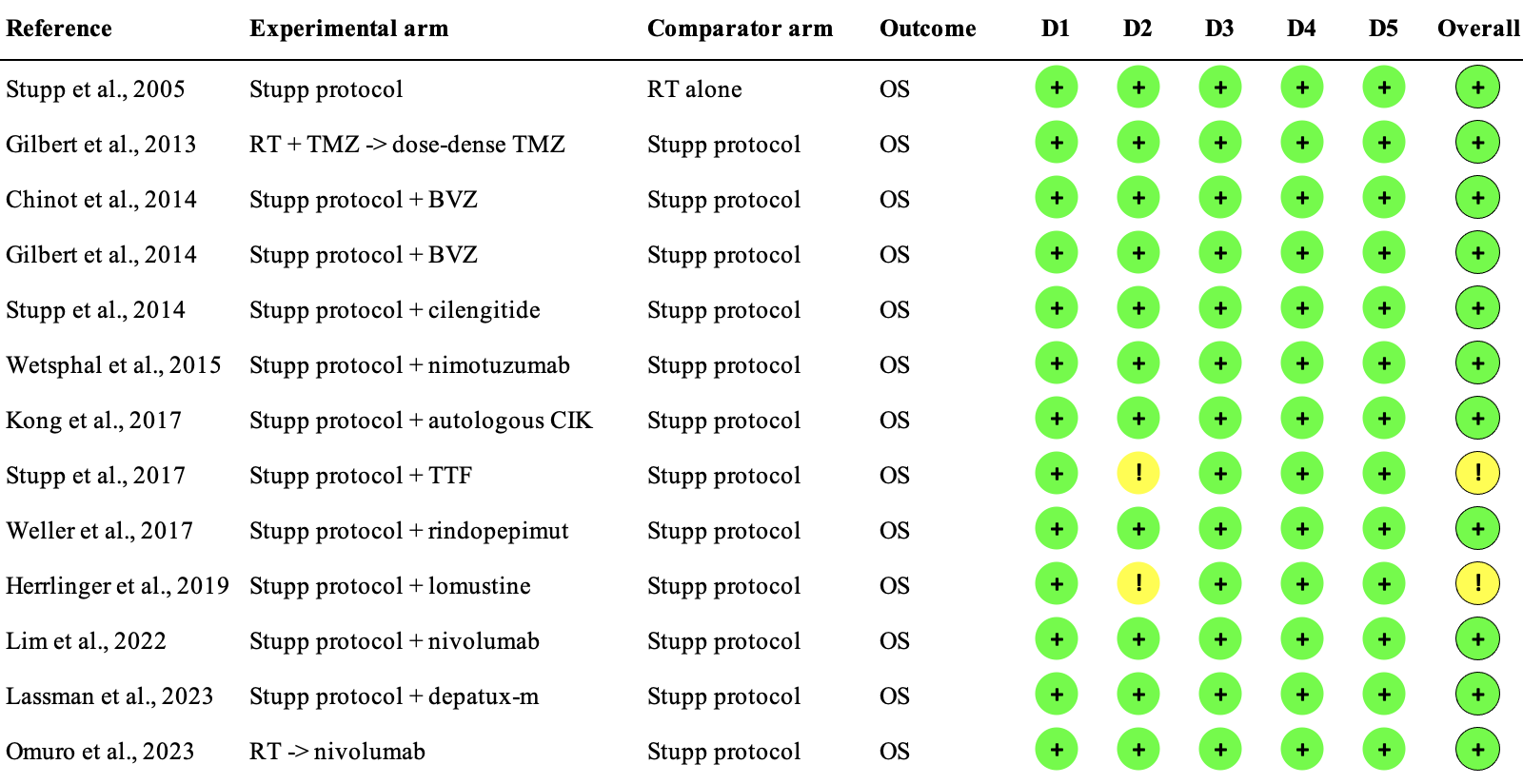
**

Adapted from <https://www.riskofbias.info/welcome/rob-2-0-tool/current-version-of-rob-2>.


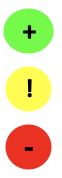

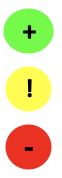

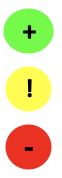
Abbreviations: D1 = randomization process, D2 = deviations from the intended interventions, D3 = missing outcome data, D4 = measurement of the outcome, D5 = selection of the reported result, RT = radiotherapy, TMZ = temozolomide, BVZ = bevacizumab, CIK = cytokine induced killer cells, TTF = tumor treating field, OS = overall survival.

= Low risk of bias, = Some concerns, = High risk of bias

**Leave-one-out analysis – Table S2**

| **Chinot et al. 2014 (AVAglio)** | | | |
| --- | --- | --- | --- |
| **Removed Study** | **N** | **Median OS** | **p value** |
| Stupp et al. 2006 | 798 | 17.7 | 0.68 |
| Gilbert et al. 2013 | 674 | 16.3 | 0.71 |
| Westphal et al. 2015 | 1014 | 16.5 | 0.54 |
| Lassman et al. 2023 | 769 | 16.3 | 0.46 |

| **Gilbert et al. 2014 (RTOG 0825)** | | | |
| --- | --- | --- | --- |
| **Removed Study** | **N** | **Median OS** | **p value** |
| Gilbert et al. 2013 | 71 | 19.1 | 0.03 |
| Westphal et al. 2015 | 411 | 16.5 | 0.2 |

Leave-one-out sensitivity analysis was conducted to investigate the impact of each individual study on the analysis. Abbreviations: N = number of patients, OS = overall survival, NA = not available.

**Comparison between reported data and reconstructed pseudodata – Table S3**

| **Author. year** | **Median OS (95% CI)** | | **Two-year OS rate (%)** | |
| --- | --- | --- | --- | --- |
|  | **Reported** | **Reconstructed** | **Reported** | **Reconstructed** |
| Stupp et al. 2005 | 14.6 (13.2 – 16.8) | 14.6 (13.2 – 16.3) | 26.5 | 26.6 |
| Gilbert et al. 2013 | 16.6 (14.9 – 18.0) | 16.5 (15.1 – 17.9) | 33.9* | 32.0 |
| Chinot et al. 2014 | 16.7 (15.4 – 18.4) | 16.6 (15.6 – 18.7) | 30 | 30.7 |
| Gilbert et al. 2014 | 16.1 (14.8 – 18.7) | 16.3 (14.9 – 18.6) | NA | 32.3 |
| Stupp et al. 2014 | 26.3 (23.9 – 34.7) | 26.8 (24.1 – 34.8) | 56 | 55.9 |
| Westphal et al. 2015 | 19.6 (14.8 – 24.0) | 19.1 (15.0 – 25.1) | NA** | 38.7 |
| Kong et al. 2017 | 16.9 (13.9 – 21.9) | 16.5 (13.9 – 25.0) | 38.5 | 37.1 |
| Stupp et al. 2017 | 16.0 (14.0 – 18.4) | 16.1 (14.1 – 18.6) | 31 | 30.6 |
| Weller et al. 2017 | 17.4 (16.1 – 19.4) | 17.6 (16.3 – 19.2) | NA | 30.0 |
| Herrlinger at al. 2019 | 31.4 (27.7 – 47.1) | 31.4 (27.8 – 47.3) | 69 | 69.8 |
| Lim et al. 2022 | 32.1 (29.4 – 33.8) | 31.9 (29.5 – 34.1) | 63.3 | 63.1 |
| Lassman et al. 2023 | 18.7 (17.0 – 20.3) | 18.9 (16.2 – 25.0) | NA | 35.9 |
| Omuro at al. 2023 | 14.9 (13.3 – 16.1) | 14.8 (13.3 – 16.1) | 21.2 | 21.3 |

*Outcomes Data From Study Registration

** The author reported a one-year survival rate of 79.1% (reconstructed: 79.2%)

Abbreviations: OS = overall survival, CI = confidence interval, NA = not available.

**Individual patient data reconstruction script**

library(meta)

library(readxl)

library(IPDfromKM)

library(survMisc)

library(survminer)

library(survHE)

library(writexl)

find_interval_limits <- function(start_time,

surv_time){

if (max(surv_time) > max(start_time))

stop("Intervals must span all survival times. Later interval missing.")

interval <- 1

end_time <- start_time[2]

upper <- NULL

for (i in seq_along(surv_time)){

if (surv_time[i] >= end_time) {

upper[interval] <- i - 1

interval <- interval + 1

end_time <- start_time[interval + 1]

}

}

cbind(lower = c(1, upper + 1),

upper = c(upper, length(surv_time)))}

#### Reconstruct Stupp protocol arms

### Chinot_2014_control

chinot_2014_control <- read_excel("~/Desktop/Lavoro/°Lavori°/Bevacizumb project/chinot_2014_control.xlsx")

interval_limits_chinot_2014_control <- find_interval_limits(start_time = c(0,3,6,9,12,15,18,21,24,27,30,33,36,39,42),

surv_time = chinot_2014_control$time)

chinot_2014_control_atrisk <- cbind (c(1,2,3,4,5,6,7,8,9,10,11,12,13,14), c (1,2,3,4,5,6,7,8,9,10,11,12,13,14), interval_limits_chinot_2014_control, c(463,444,405,355,293,245,201,163,118,84,53,26,15,6))

colnames (chinot_2014_control_atrisk) [2] <- "Interval"

colnames (chinot_2014_control_atrisk) [5] <- "nrisk"

chinot_2014_control_data <- cbind("k" = rownames(chinot_2014_control), chinot_2014_control)

write.table(chinot_2014_control_atrisk, file = "chinot_2014_control_atrisk.txt", row.names = FALSE)

write.table(chinot_2014_control_data, file = "chinot_2014_control_data.txt", row.names = FALSE)

digitise(surv_inp = "chinot_2014_control_data.txt",nrisk_inp = "chinot_2014_control_atrisk.txt")

IPDdata <- read.table("IPDdata.txt", header = TRUE)

KMdata <- read.table("KMdata.txt", header = TRUE)

KM.est <- survfit(Surv(time, event) ~ 1, data = IPDdata, type = "kaplan-meier",)

surv_median(KM.est)

IPD <- as.data.frame(IPDdata)

write_xlsx(IPD,"~/Desktop/Lavoro/°Lavori°/Bevacizumb project/chinot_2014_control_IPD.xlsx")

### Gilbert_2013

gilbert_2013 <- read_excel("~/Desktop/Lavoro/°Lavori°/Bevacizumb project/gilbert_2013.xlsx")

interval_limits_gilbert_2013 <- find_interval_limits(start_time = c(0,12,24,36,48),

surv_time = gilbert_2013$time)

gilbert_2013_atrisk <- cbind (c(1,2,3,4), c (1,2,3,4), interval_limits_gilbert_2013, c(411,257,121,32))

colnames (gilbert_2013_atrisk) [2] <- "Interval"

colnames (gilbert_2013_atrisk) [5] <- "nrisk"

gilbert_2013_data <- cbind("k" = rownames(gilbert_2013), gilbert_2013)

write.table(gilbert_2013_atrisk, file = "gilbert_2013_atrisk.txt", row.names = FALSE)

write.table(gilbert_2013_data, file = "gilbert_2013_data.txt", row.names = FALSE)

digitise(surv_inp = "gilbert_2013_data.txt",nrisk_inp = "gilbert_2013_atrisk.txt")

IPDdata <- read.table("IPDdata.txt", header = TRUE)

KMdata <- read.table("KMdata.txt", header = TRUE)

KM.est <- survfit(Surv(time, event) ~ 1, data = IPDdata, type = "kaplan-meier",)

surv_median(KM.est)

IPD <- as.data.frame(IPDdata)

write_xlsx(IPD,"~/Desktop/Lavoro/°Lavori°/Bevacizumb project/gilbert_2013_IPD.xlsx")

### Gilbert_2014_control arm

gilbert_2014_control <- read_excel("~/Desktop/Lavoro/°Lavori°/Bevacizumb project/gilbert_2014_control.xlsx")

interval_limits_gilbert_2014_control <- find_interval_limits(start_time = c(0,6,12,18,24,30,31),

surv_time = gilbert_2014_control$time)

gilbert_2014_control_atrisk <- cbind (c(1,2,3,4,5,6), c (1,2,3,4,5,6), interval_limits_gilbert_2014_control, c(309,255,192,112,50,22))

colnames (gilbert_2014_control_atrisk) [2] <- "Interval"

colnames (gilbert_2014_control_atrisk) [5] <- "nrisk"

gilbert_2014_control_data <- cbind("k" = rownames(gilbert_2014_control), gilbert_2014_control)

write.table(gilbert_2014_control_atrisk, file = "gilbert_2014_control_atrisk.txt", row.names = FALSE)

write.table(gilbert_2014_control_data, file = "gilbert_2014_control_data.txt", row.names = FALSE)

digitise(surv_inp = "gilbert_2014_control_data.txt",nrisk_inp = "gilbert_2014_control_atrisk.txt")

IPDdata <- read.table("IPDdata.txt", header = TRUE)

KMdata <- read.table("KMdata.txt", header = TRUE)

KM.est <- survfit(Surv(time, event) ~ 1, data = IPDdata, type = "kaplan-meier",)

surv_median(KM.est)

IPD <- as.data.frame(IPDdata)

write_xlsx(IPD,"~/Desktop/Lavoro/°Lavori°/Bevacizumb project/gilbert_2014_control_IPD.xlsx")

### Herrlinger 2019

herrlinger_2019 <- read_excel("~/Desktop/Lavoro/°Lavori°/Bevacizumb project/herrlinger_2019.xlsx")

interval_limits_herrlinger_2019 <- find_interval_limits(start_time = c(0,12,24,36,48,60,72),

surv_time = herrlinger_2019$time)

herrlinger_2019_atrisk <- cbind (c(1,2,3,4,5,6), c (1,2,3,4,5,6), interval_limits_herrlinger_2019, c(60,49,38,23,11,1))

colnames (herrlinger_2019_atrisk) [2] <- "Interval"

colnames (herrlinger_2019_atrisk) [5] <- "nrisk"

herrlinger_2019_data <- cbind("k" = rownames(herrlinger_2019), herrlinger_2019)

write.table(herrlinger_2019_atrisk, file = "herrlinger_2019_atrisk.txt", row.names = FALSE)

write.table(herrlinger_2019_data, file = "herrlinger_2019_data.txt", row.names = FALSE)

digitise(surv_inp = "herrlinger_2019_data.txt",nrisk_inp = "herrlinger_2019_atrisk.txt")

IPDdata <- read.table("IPDdata.txt", header = TRUE)

KMdata <- read.table("KMdata.txt", header = TRUE)

KM.est <- survfit(Surv(time, event) ~ 1, data = IPDdata, type = "kaplan-meier",)

surv_median(KM.est)

IPD <- as.data.frame(IPDdata)

write_xlsx(IPD,"~/Desktop/Lavoro/°Lavori°/Bevacizumb project/herrlinger_2019_IPD.xlsx")

### Kong 2017

kong_2017 <- read_excel("~/Desktop/Lavoro/°Lavori°/Bevacizumb project/kong_2017.xlsx")

interval_limits_kong_2017 <- find_interval_limits(start_time = c(0,10,20,30, 40,50),

surv_time = kong_2017$time)

kong_2017_atrisk <- cbind (c(1,2,3,4,5), c (1,2,3,4,5), interval_limits_kong_2017, c(89,69,26,13,3))

colnames (kong_2017_atrisk) [2] <- "Interval"

colnames (kong_2017_atrisk) [5] <- "nrisk"

kong_2017_data <- cbind("k" = rownames(kong_2017), kong_2017)

write.table(kong_2017_atrisk, file = "kong_2017_atrisk.txt", row.names = FALSE)

write.table(kong_2017_data, file = "kong_2017_data.txt", row.names = FALSE)

digitise(surv_inp = "kong_2017_data.txt",nrisk_inp = "kong_2017_atrisk.txt")

IPDdata <- read.table("IPDdata.txt", header = TRUE)

KMdata <- read.table("KMdata.txt", header = TRUE)

KM.est <- survfit(Surv(time, event) ~ 1, data = IPDdata, type = "kaplan-meier",)

surv_median(KM.est)

IPD <- as.data.frame(IPDdata)

write_xlsx(IPD,"~/Desktop/Lavoro/°Lavori°/Bevacizumb project/kong_2017_IPD.xlsx")

### Lassman 2023

lassman_2023 <- read_excel("~/Desktop/Lavoro/°Lavori°/Bevacizumb project/lassman_2023.xlsx")

interval_limits_lassman_2023 <- find_interval_limits(start_time = c(0,6,12,18,24,30),

surv_time = lassman_2023$time)

lassman_2023_atrisk <- cbind (c(1,2,3,4,5), c (1,2,3,4,5), interval_limits_lassman_2023, c(316,144,63,29,12))

colnames (lassman_2023_atrisk) [2] <- "Interval"

colnames (lassman_2023_atrisk) [5] <- "nrisk"

lassman_2023_data <- cbind("k" = rownames(lassman_2023), lassman_2023)

write.table(lassman_2023_atrisk, file = "lassman_2023_atrisk.txt", row.names = FALSE)

write.table(lassman_2023_data, file = "lassman_2023_data.txt", row.names = FALSE)

digitise(surv_inp = "lassman_2023_data.txt",nrisk_inp = "lassman_2023_atrisk.txt")

IPDdata <- read.table("IPDdata.txt", header = TRUE)

KMdata <- read.table("KMdata.txt", header = TRUE)

KM.est <- survfit(Surv(time, event) ~ 1, data = IPDdata, type = "kaplan-meier",)

surv_median(KM.est)

IPD <- as.data.frame(IPDdata)

write_xlsx(IPD,"~/Desktop/Lavoro/°Lavori°/Bevacizumb project/lassman_2023_IPD.xlsx")

### Lim 2022

lim_2022 <- read_excel("~/Desktop/Lavoro/°Lavori°/Bevacizumb project/lim_2022.xlsx")

interval_limits_lim_2022 <- find_interval_limits(start_time = c(0,6,12,18,24,30,36,42,48,54),

surv_time = lim_2022$time)

lim_2022_atrisk <- cbind (c(1,2,3,4,5,6,7,8,9), c (1,2,3,4,5,6,7,8,9), interval_limits_lim_2022, c(358, 335,304, 260, 214, 166, 81,43,9))

colnames (lim_2022_atrisk) [2] <- "Interval"

colnames (lim_2022_atrisk) [5] <- "nrisk"

lim_2022_data <- cbind("k" = rownames(lim_2022), lim_2022)

write.table(lim_2022_atrisk, file = "lim_2022_atrisk.txt", row.names = FALSE)

write.table(lim_2022_data, file = "lim_2022_data.txt", row.names = FALSE)

digitise(surv_inp = "lim_2022_data.txt",nrisk_inp = "lim_2022_atrisk.txt")

IPDdata <- read.table("IPDdata.txt", header = TRUE)

KMdata <- read.table("KMdata.txt", header = TRUE)

KM.est <- survfit(Surv(time, event) ~ 1, data = IPDdata, type = "kaplan-meier",)

surv_median(KM.est)

IPD <- as.data.frame(IPDdata)

write_xlsx(IPD,"~/Desktop/Lavoro/°Lavori°/Bevacizumb project/lim_2022_IPD.xlsx")

### Omuro 2023

omuro_2023 <- read_excel("~/Desktop/Lavoro/°Lavori°/Bevacizumb project/omuro_2023.xlsx")

interval_limits_omuro_2023 <- find_interval_limits(start_time = c(0,3, 6, 9, 12, 15, 18, 21, 24, 27 ,30 ,33 ),

surv_time = omuro_2023$time)

omuro_2023_atrisk <- cbind (c(1,2,3,4,5,6,7,8,9,10,11), c (1,2,3,4,5,6,7,8,9,10,11), interval_limits_omuro_2023, c(280, 272, 242, 212, 166, 131, 92, 67, 37, 19, 2))

colnames (omuro_2023_atrisk) [2] <- "Interval"

colnames (omuro_2023_atrisk) [5] <- "nrisk"

omuro_2023_data <- cbind("k" = rownames(omuro_2023), omuro_2023)

write.table(omuro_2023_atrisk, file = "omuro_2023_atrisk.txt", row.names = FALSE)

write.table(omuro_2023_data, file = "omuro_2023_data.txt", row.names = FALSE)

digitise(surv_inp = "omuro_2023_data.txt",nrisk_inp = "omuro_2023_atrisk.txt")

IPDdata <- read.table("IPDdata.txt", header = TRUE)

KMdata <- read.table("KMdata.txt", header = TRUE)

KM.est <- survfit(Surv(time, event) ~ 1, data = IPDdata, type = "kaplan-meier",)

surv_median(KM.est)

IPD <- as.data.frame(IPDdata)

write_xlsx(IPD,"~/Desktop/Lavoro/°Lavori°/Bevacizumb project/omuro_2023_IPD.xlsx")

### Stupp 2005

stupp_2005 <- read_excel("~/Desktop/Lavoro/°Lavori°/Bevacizumb project/stupp_2005.xlsx")

interval_limits_stupp_2005 <- find_interval_limits(start_time = c(0,6,12,18,24,30,36,42),

surv_time = stupp_2005$time)

stupp_2005_atrisk <- cbind (c(1,2,3,4,5,6,7), c (1,2,3,4,5,6,7), interval_limits_stupp_2005, c(287, 246, 174, 109, 57,27,4 ))

colnames (stupp_2005_atrisk) [2] <- "Interval"

colnames (stupp_2005_atrisk) [5] <- "nrisk"

stupp_2005_data <- cbind("k" = rownames(stupp_2005), stupp_2005)

write.table(stupp_2005_atrisk, file = "stupp_2005_atrisk.txt", row.names = FALSE)

write.table(stupp_2005_data, file = "stupp_2005_data.txt", row.names = FALSE)

digitise(surv_inp = "stupp_2005_data.txt",nrisk_inp = "stupp_2005_atrisk.txt")

IPDdata <- read.table("IPDdata.txt", header = TRUE)

KMdata <- read.table("KMdata.txt", header = TRUE)

KM.est <- survfit(Surv(time, event) ~ 1, data = IPDdata, type = "kaplan-meier",)

surv_median(KM.est)

IPD <- as.data.frame(IPDdata)

write_xlsx(IPD,"~/Desktop/Lavoro/°Lavori°/Bevacizumb project/stupp_2005_IPD.xlsx")

### Stupp 2014

stupp_2014 <- read_excel("~/Desktop/Lavoro/°Lavori°/Bevacizumb project/stupp_2014.xlsx")

interval_limits_stupp_2014 <- find_interval_limits(start_time = c(0, 3, 6, 9, 12, 15, 18 ,21, 24, 27 ,30 ,33 , 36, 39 , 42, 45, 48),

surv_time = stupp_2014$time)

stupp_2014_atrisk <- cbind (c(1,2,3,4,5,6,7,8,9,10,11,12,13,14,15,16), c (1,2,3,4,5,6,7,8,9,10,11,12,13,14,15,16), interval_limits_stupp_2014, c(273,259, 242,221,212,196, 172, 155, 120, 80, 58,40, 24,14,5,1))

colnames (stupp_2014_atrisk) [2] <- "Interval"

colnames (stupp_2014_atrisk) [5] <- "nrisk"

stupp_2014_data <- cbind("k" = rownames(stupp_2014), stupp_2014)

write.table(stupp_2014_atrisk, file = "stupp_2014_atrisk.txt", row.names = FALSE)

write.table(stupp_2014_data, file = "stupp_2014_data.txt", row.names = FALSE)

digitise(surv_inp = "stupp_2014_data.txt",nrisk_inp = "stupp_2014_atrisk.txt")

IPDdata <- read.table("IPDdata.txt", header = TRUE)

KMdata <- read.table("KMdata.txt", header = TRUE)

KM.est <- survfit(Surv(time, event) ~ 1, data = IPDdata, type = "kaplan-meier",)

surv_median(KM.est)

IPD <- as.data.frame(IPDdata)

write_xlsx(IPD,"~/Desktop/Lavoro/°Lavori°/Bevacizumb project/stupp_2014_IPD.xlsx")

### Stupp 2017

stupp_2017 <- read_excel("~/Desktop/Lavoro/°Lavori°/Bevacizumb project/stupp_2017.xlsx")

interval_limits_stupp_2017 <- find_interval_limits(start_time = c(0, 6, 12, 18, 24, 30, 36, 42, 48, 54, 60),

surv_time = stupp_2017$time)

stupp_2017_atrisk <- cbind (c(1,2,3,4,5,6,7,8,9,10), c (1,2,3,4,5,6,7,8,9,10), interval_limits_stupp_2017, c(229, 191, 144, 95, 60, 33, 22, 13, 7, 5))

colnames (stupp_2017_atrisk) [2] <- "Interval"

colnames (stupp_2017_atrisk) [5] <- "nrisk"

stupp_2017_data <- cbind("k" = rownames(stupp_2017), stupp_2017)

write.table(stupp_2017_atrisk, file = "stupp_2017_atrisk.txt", row.names = FALSE)

write.table(stupp_2017_data, file = "stupp_2017_data.txt", row.names = FALSE)

digitise(surv_inp = "stupp_2017_data.txt",nrisk_inp = "stupp_2017_atrisk.txt")

IPDdata <- read.table("IPDdata.txt", header = TRUE)

KMdata <- read.table("KMdata.txt", header = TRUE)

KM.est <- survfit(Surv(time, event) ~ 1, data = IPDdata, type = "kaplan-meier",)

surv_median(KM.est)

IPD <- as.data.frame(IPDdata)

write_xlsx(IPD,"~/Desktop/Lavoro/°Lavori°/Bevacizumb project/stupp_2017_IPD.xlsx"

### Westphal 2015

westphal_2015 <- read_excel("~/Desktop/Lavoro/°Lavori°/Bevacizumb project/westphal_2015.xlsx")

interval_limits_westphal_2015 <- find_interval_limits(start_time = c(0,6,12,18,24,30,36,42),

surv_time = westphal_2015$time)

westphal_2015_atrisk <- cbind (c(1,2,3,4,5,6,7), c (1,2,3,4,5,6,7), interval_limits_westphal_2015, c(71,66,51,31,17,11,3))

colnames (westphal_2015_atrisk) [2] <- "Interval"

colnames (westphal_2015_atrisk) [5] <- "nrisk"

westphal_2015_data <- cbind("k" = rownames(westphal_2015), westphal_2015)

write.table(westphal_2015_atrisk, file = "westphal_2015_atrisk.txt", row.names = FALSE)

write.table(westphal_2015_data, file = "westphal_2015_data.txt", row.names = FALSE)

digitise(surv_inp = "westphal_2015_data.txt",nrisk_inp = "westphal_2015_atrisk.txt")

IPDdata <- read.table("IPDdata.txt", header = TRUE)

KMdata <- read.table("KMdata.txt", header = TRUE)

KM.est <- survfit(Surv(time, event) ~ 1, data = IPDdata, type = "kaplan-meier",)

surv_median(KM.est)

IPD <- as.data.frame(IPDdata)

write_xlsx(IPD,"~/Desktop/Lavoro/°Lavori°/Bevacizumb project/westphal_2015_IPD.xlsx")

### Weller 2017

weller_2017 <- read_excel("~/Desktop/Lavoro/°Lavori°/Bevacizumb project/weller_2017.xlsx")

interval_limits_weller_2017 <- find_interval_limits(start_time = c(0, 6, 12, 18, 24, 30, 36, 42, 48),

surv_time = weller_2017$time)

weller_2017_atrisk <- cbind (c(1,2,3,4,5,6,7,8), c (1,2,3,4,5,6,7,8), interval_limits_weller_2017, c(374,347, 268, 149, 73, 25,8, 4))

colnames (weller_2017_atrisk) [2] <- "Interval"

colnames (weller_2017_atrisk) [5] <- "nrisk"

weller_2017_data <- cbind("k" = rownames(weller_2017), weller_2017)

write.table(weller_2017_atrisk, file = "weller_2017_atrisk.txt", row.names = FALSE)

write.table(weller_2017_data, file = "weller_2017_data.txt", row.names = FALSE)

digitise(surv_inp = "weller_2017_data.txt",nrisk_inp = "weller_2017_atrisk.txt")

IPDdata <- read.table("IPDdata.txt", header = TRUE)

KMdata <- read.table("KMdata.txt", header = TRUE)

KM.est <- survfit(Surv(time, event) ~ 1, data = IPDdata, type = "kaplan-meier",)

surv_median(KM.est)

IPD <- as.data.frame(IPDdata)

write_xlsx(IPD,"~/Desktop/Lavoro/°Lavori°/Bevacizumb project/weller_2017_IPD.xlsx")

#### bevacizumab arms

### Chinot 2014_bevacizumab arm

chinot_2014_beva <- read_excel("~/Desktop/Lavoro/°Lavori°/Bevacizumb project/chinot_2014_beva.xlsx")

interval_limits_chinot_2014_beva <- find_interval_limits(start_time = c(0,3,6,9,12,15,18,21,24,27,30,33,36,39,42,45),

surv_time = chinot_2014_beva$time)

chinot_2014_beva_atrisk <- cbind (c(1,2,3,4,5,6,7,8,9,10,11,12,13,14,15), c (1,2,3,4,5,6,7,8,9,10,11,12,13,14,15), interval_limits_chinot_2014_beva, c(458,440,421,387,322,253,203,176,139,91,61,27,11,4,1))

colnames (chinot_2014_beva_atrisk) [2] <- "Interval"

colnames (chinot_2014_beva_atrisk) [5] <- "nrisk"

chinot_2014_beva_data <- cbind("k" = rownames(chinot_2014_beva), chinot_2014_beva)

write.table(chinot_2014_beva_atrisk, file = "chinot_2014_beva_atrisk.txt", row.names = FALSE)

write.table(chinot_2014_beva_data, file = "chinot_2014_beva_data.txt", row.names = FALSE)

digitise(surv_inp = "chinot_2014_beva_data.txt",nrisk_inp = "chinot_2014_beva_atrisk.txt")

IPDdata <- read.table("IPDdata.txt", header = TRUE)

KMdata <- read.table("KMdata.txt", header = TRUE)

KM.est <- survfit(Surv(time, event) ~ 1, data = IPDdata, type = "kaplan-meier",)

surv_median(KM.est)

IPD <- as.data.frame(IPDdata)

write_xlsx(IPD,"~/Desktop/Lavoro/°Lavori°/Bevacizumb project/chinot_2014_beva_IPD.xlsx")

### Gilbert 2014_bevacizuamb arm

gilbert_2014_beva <- read_excel("~/Desktop/Lavoro/°Lavori°/Bevacizumb project/gilbert_2014_beva.xlsx")

interval_limits_gilbert_2014_beva <- find_interval_limits(start_time = c(0,6,12,18,24,30,31),

surv_time = gilbert_2014_beva$time)

gilbert_2014_beva_atrisk <- cbind (c(1,2,3,4,5,6), c (1,2,3,4,5,6), interval_limits_gilbert_2014_beva, c(312,263,200, 99,47,17))

colnames (gilbert_2014_beva_atrisk) [2] <- "Interval"

colnames (gilbert_2014_beva_atrisk) [5] <- "nrisk"

gilbert_2014_beva_data <- cbind("k" = rownames(gilbert_2014_beva), gilbert_2014_beva)

write.table(gilbert_2014_beva_atrisk, file = "gilbert_2014_beva_atrisk.txt", row.names = FALSE)

write.table(gilbert_2014_beva_data, file = "gilbert_2014_beva_data.txt", row.names = FALSE)

digitise(surv_inp = "gilbert_2014_beva_data.txt",nrisk_inp = "gilbert_2014_beva_atrisk.txt")

IPDdata <- read.table("IPDdata.txt", header = TRUE)

KMdata <- read.table("KMdata.txt", header = TRUE)

KM.est <- survfit(Surv(time, event) ~ 1, data = IPDdata, type = "kaplan-meier",)

surv_median(KM.est)

IPD <- as.data.frame(IPDdata)

write_xlsx(IPD,"~/Desktop/Lavoro/°Lavori°/Bevacizumb project/gilbert_2014_beva_IPD.xlsx")
